# Martin Buber: guide for a psychology of suffering

**DOI:** 10.3389/fpsyg.2023.1154865

**Published:** 2023-05-12

**Authors:** Roger G. Tweed, Thomas P. Bergen, Kristina K. Castaneto, Andrew G. Ryder

**Affiliations:** ^1^Department of Psychology, Douglas College, New Westminster, BC, Canada; ^2^Department of Psychology, Kwantlen Polytechnic University, Surrey, BC, Canada; ^3^Department of Religion and Theology, Vrije Universiteit, Amsterdam, Netherlands; ^4^Centre for Clinical Research in Health and Department of Psychology, Concordia University, Montréal, QC, Canada; ^5^Culture and Mental Health Research Unit and Lady Davis Institute, Jewish General Hospital, Montréal, QC, Canada

**Keywords:** Martin Buber, dialogue, suffering, guide, psychology, positive psychology

## Abstract

Martin Buber was untrained in psychology, yet his teaching provides helpful guidance for a psychological science of suffering. His ideas deserve attention at three distinct levels. For each of these, his ideas align with research findings, but also push beyond them. At the individual level, Buber’s radical approach to relationships disrupts typical social cognitive cycles of suffering and can thereby build a defense against suffering. At the community level, he provides guidance that can help create a society that cares for people who suffer. At the dyadic level, Buber’s guidance also matters. His ideas point toward a therapeutic dyad that can help address suffering when the individual and community responses are not sufficient. Specifically, he guides us toward a holistic view of the person that transcends labels and also toward ineffable human relations. Here again, his ideas align with empirical research, but push beyond. Buber’s unique take on relationships has much to offer scholars seeking to understand and alleviate suffering. Some might perceive Buber as ignoring evil. That possible criticism and others deserve consideration. Nonetheless, readiness to adjust theory in response to Buber and other psychological outsiders may be valuable when developing a psychology of suffering.

## 1. Introduction

This collection of articles is devoted to developing a psychology of suffering, a field of study clarifying the nature of suffering and offering paths for transcending suffering and building a good life. Martin Buber’s ideas deserve consideration here because suffering was central to his thought. Friedman (2013) concluded, after a lifetime that included both friendship with Buber and intensive study of his works, that “the innermost core of Buber’s teaching” is his attitude toward suffering (p. 163).

Nonetheless, why do we need a *psychology of suffering* in a discipline that already devotes considerable attention to psychopathology, along with clinical and counseling psychology? After all, much psychological research already focuses on negative aspects of human life, including various forms of suffering.

Indeed, near the beginning of this century, the ‘positive psychologists’ argued that psychological research was already saturated with negativity (Seligman, 1998a,b; Gable and Haidt, 2005). According to these critics, psychologists had failed to study and promote the good life—a striking neglect, as people need much more than mere alleviation of suffering. Positive psychologists responded by studying the good life and developing interventions to induce happiness (Seligman et al., 2005). Some of their strategies seemed to work well (e.g., Seligman et al., 2005). Interventions included exercises such as focusing on gratitude, or focusing on one’s strengths, or considering one’s ideal self.

However, positive psychology’s effort to bring balance created an equal but opposite imbalance: a focus on happiness to the exclusion of the need to address suffering (Wong, 2011). A call for people to be happy or even happiness interventions can be premature or even depressing if one is in the midst of suffering. Some empirical evidence supported this notion: brief happiness-inducing positive psychology strategies performed poorly for people who seemed to have the greatest need (Lyubomirsky et al., 2011).

Wong (2023a) has called for a new positive psychology, an existential positive psychology, built on a realistic presumption that all people face suffering, that suffering can be accepted, and that a willingness to face and transcend suffering is part of the foundation of a deep and stable form of well-being. From this perspective (Wong, 2023a), the good life requires facing and balancing both the light and dark side of life. He suggests that suffering is not something to flee, but instead something to face and transcend, so one can also turn toward and experience the positive aspects of life. Many sources of suffering exist such as chronic disease, genetic predisposition to lower levels of cheerfulness (Tov et al., 2022), and structural societal barriers, so suffering is pervasive. According to existential positive psychology (Wong, 2022), transcending suffering involves going beyond your individual self and contributing to humanity. Thus, existential positive psychology is relational. The relations Wong (2023b) highlights include not only relations within the self, but also relations with others, and with a faith world. Buber’s orientation, as discussed in the current manuscript, focuses on relations with others as a response to a world in which one suffers, so, in some sense, Wong’s perspective has links with Buber’s ideas.

This discussion introduces Buber’s central ideas while also illustrating their relevance to a psychology that helps people transcend suffering. We discuss implications in three levels: individual, community, and dyadic-therapeutic. At the individual level, Buber advocates for patterns of being that counter cognitive cycles of suffering. At the community level, his ideas address the separation, loneliness, and alienation that accompanies much suffering. At the dyadic-therapeutic level, Buber has relevance for an ineffable bond that transcends disconnection. In discussing professional therapy, we will also discuss his holistic view of the person and its relevance to diagnostic labels. At each of these points, Buber charts a path toward healthy responses to suffering, a path toward a good life that does not ignore the reality of suffering. Our overarching argument is that, for each of these levels, Buber’s ideas fit with relevant contemporary research while also pointing beyond.

## 2. Who was Martin Buber?

Born in Vienna in 1878, Martin Buber’s parents divorced when he was only 3 years of age, so he was raised by his grandfather in the city now known as Lviv, in Ukraine. Although raised in an Orthodox Jewish environment, a religious crisis in adolescence led him to abandon traditional practice and begin studies in philosophy. In 1896, he returned to Vienna to pursue formal studies in the discipline. After periods spent in Berlin and Heppenheim in Germany, Buber moved to Frankfurt in 1930 to take up a professorship—but he resigned in protest immediately after the rise of Adolf Hitler in 1933. Five years later, he left Germany and settled in Jerusalem, where he spent the rest of his life until his death in 1965 (Friedman, 2013; Zank and Braiterman, 2020).

Buber’s early intellectual work consisted largely of mystical and mythical—most famously, Hasidic—texts grounded in predominantly Jewish sources. Although he had not yet set down a personal philosophy, the seeds of later concerns with dialogue and connection can already be observed in these works. His most famous book, *Ich und Du* (hereafter, “I and Thou”) was published in 1923. Not long after, he undertook a translation of the Hebrew Bible into German in collaboration with Franz Rosenzweig, a translation known for its innovative phrasing designed to emphasize the multiple meanings inherent in the Hebrew original (Kaufmann, 1970).

In his later years, Buber became the most well-known Israeli professor of his time. He increasingly participated in political activism, including support for the goal of a bi-national state for Arabs and Jews in Israel/Palestine. This period also brought with it more engagement with psychiatry and psychology, including published correspondence with Carl Jung and Carl Rogers (Buber Agassi, 1999). Some of this work was influential to the founders of existential psychotherapy during the 1950s and 1960s, in particular the attention to the uniqueness of each individual person and the centrality of human connection between individual people (Roazen, 1999). In the pages that follow, we consider the continued relevance of Buber’s I and Thou, as interpreted through the lens of his engagement with the psy-disciplines, to contemporary psychologists.

## 3. I-Thou versus I-It: construed, turned, exclusive, present, unbounded, reverent, impermanent, and transforming

We’ve used eight descriptors to summarize the Buber’s central concept of an I-Thou relation: construed, turned, exclusive, present, unbounded, reverent, impermanent, and transforming. If people know only one idea from Buber, it is his distinction between “I-It” and “I-Thou.” Buber (1958) suggested that one can relate to others in two ways: I-It or I-Thou. As a person walks through their day, they will have many I-It interactions; they may also have some I-Thou interactions. The distinction between these two, he suggests, is fundamental to human experience. A person can consider their day, and if they understand the nature of this distinction, they can judge each moment of interaction to be more I-It or more I-Thou. Without denying that many interactions would necessarily take an I-It form even over the course of a close relationship, Buber consistently advocated for more I-Thou.

Why “Thou”? Terms such as “intimate you” or “dear one” would capture part of Buber’s idea of “thou.” To the English-hearing ear, “thou” sounds formal and reverent, but *thou* was originally singular, informal, and intimate. This distinction remains in the original German—the book’s original title is *Ich und Du*, not *Ich und Sie*—and exists in many other languages (e.g., French tu vs. vous). The greater intimacy of Du/Thou is central to Buber’s idea, but no single English word captures this sense. The change in connotation for the English ‘thou’ over the centuries poses a translation challenge. As Kaufmann (1970, p. 14) notes in the introduction to his translation of ‘I and Thou’, “Thou and you are not the same. Nor is Thou very similar to the German *Du*. German lovers say *Du* to one another, and so do friends. *Du* is spontaneous and unpretentious, remote from formality, pomp, and dignity.” English lovers do not address each other as thou. Interestingly, the Quakers held on to the use of ‘thou’ for a long time, precisely to retain this distinction so that free and equal individual people would be able to address each other as familiar and intimate—and would similarly address God.

### 3.1. Construed

An I-Thou relation involves construal. In the domain of the senses, perceptions may be correct or incorrect: if you think you see the sun rise, then you are either right or wrong. Construal is different. Construal, as frequently used by social psychologists, suggests some arbitrariness in the nature of the perception. When perceiving a person as thou or it, the perception depends not only on the person being perceived, but on the orientation of the perceiver. If you perceive (or construe) your employer to be dishonest, that partially determines the relationship. If you perceive your interlocutor to be thou, your experience will change. The self has some power to influence whether the interaction will be I-Thou or I-It.

### 3.2. Turned

I-Thou involves turning, in a deep relational sense, toward the other person “with the intention of establishing a living mutual relation” between yourself and them and an openness to being influenced by the other person (Buber, 2004). Buber wrote, “You, imprisoned in the shells in which society, state, church, school, economy, public opinion, and your own pride have stuck you, indirect one among indirect ones, break through your shells, become direct; man [and woman], have contact with men [and women]!” (Buber, 1957, p. 109). A reciprocal I-Thou relation requires the other to likewise turn, but one can initiate a form of I-Thou regardless of the participation of the other (Buber, 1970).

### 3.3. Exclusive

Buber suggested that other concerns recede if you turn toward another person and construe them as thou. You still perceive other aspects of the world, time of day, other sounds, but you perceive these other aspects of the world in relation to or in light of this other person. You are not distracted from this person with whom you are in dialogue. As Buber said, “Everything else can only be background” (Buber, 1970, p. 80). Thinking of the other as a Thou, is an act of “one’s whole being” (Buber, 1970, p. 61), and thus, you lack mental capacity to focus on other factors. This exclusivity is perhaps easiest to grasp in the negative. If other events, past or future, or other concerns distract you, then you are not experiencing I-Thou.

### 3.4. Present

You focus on the present. If you instead decided to think in a strategic, even Machiavellian way in order to gain more power, you would think about what you have learned in the past to secure a better future. If you are seeking to sell the person an idea in order to gain a commission, to gain respect, or to gain a convert, you are focused on the future rather than the present. I-Thou directs you instead to the present.

### 3.5. Unbounded

According to Buber, treating a person as an it, involves limits and boundaries. The person is a thing amidst other things (Buber, 1958, p. 13). For example, using labels places boundaries; they place others in a class of people. A person may be kind or a cheat or a teammate or a sufferer from schizophrenia, but within an I-Thou relation, the I is conscious that the other is also much more. For Buber, even admiration degrades the other into an It, as it brings the danger of focusing on something specific such as their beauty, intelligence, virtue, or something else. It treats the person as a “bundle of named qualities” (Buber, 1958, p. 13). In contrast, a thou, suggests Buber, fills the heavens.

### 3.6. Reverent

Buber’s exhortation to treat the other as somewhat mysterious begins to border on reverence (Adame and Leitner, 2011). Within religious and spiritual contexts, there are longstanding traditions treating valued ideals and entities as mysterious and somewhat unknowable. Arguments for negative theology are seen among some medieval Jewish theologians, such as Maimonides in *Guide of the Perplexed* (Ivry, 2016), some Eastern Orthodox Christian thinkers (Lossky, 1976), and some Western mystical authors such as the author of the *Cloud of Unknowing* (Wolters, 2001). They suggested that the best way to define the mysterious God is to assert what God is not; God is not human, God is not bound by time, and God is not limited by location, etc. God is ineffable. This approach to theology maintains mystery, and parallels Buber’s approach to Thou: as precious, undefinable, uncontained, and even as sacred. Indeed, he explicitly makes this connection to God in Part 3 of I and Thou (Buber, 1970).

### 3.7. Impermanent

No one permanently persists in pure I-Thou relation. One must shop; one must make other exchanges in the marketplace to win work and gain security. In daily coordination with one’s romantic partner, one may need to arrange chores and school pick-up schedules. Nonetheless, I-Thou moments can occur and can be transformative. But after the I-Thou moment passes, Buber suggested that a person can still retain a spark such that the I-It world presses less heavily on the self (Buber, 1958, p. 45).

### 3.8. Transforming

Nihilists deny that any greater meaning to life can be known because they think no meaning exists. Buber argued something similar, but importantly distinct; he believed the meta-narrative may exist, but is often hidden and unknowable (Buber, 2016). The apparent lack of meaning and purpose can be distressing (Wong, 2012; George and Park, 2017). Buber believed that some people, in the midst of this feeling of meaninglessness, can turn toward others and treat them as thou. Buber believed that the person who turns then crystalizes toward the form of their true self. Kramer (2019), a devotee of Buber, summarized this position well: “the innermost growth of the self, despite what many people say, does not come in relation to one’s self. Rather attaining one’s authentic human existence emerges again and again through dialogue or in the realms of participatory consciousness” (loc. 207). Buber expected differences between people to persist. They would not become the same as each other—indeed, as they engage in I-Thou relations, they would become even more distinct—but they would each become their true unique self.

People regularly embodying Buber’s ideals can thus seem strange because they become a different type of person. Francis of Assisi had no governmental position, but he valued relationships and people, and decided to stop a war by going to talk to the leader of the other side (Chesterton, 1990). He saw no reason to believe that the leader of his enemies was not an individual worthy of dialogue. Francis’ demeanor seemed so bizarre that the leader seems to have simply let Francis walk free. Likewise, Coretta Scott King (King and King, 2010) reported that civil rights activists of 1960’s America were distinguished by their belief that some of their opponents might respond to moral suasion, that they might change their hearts. They presumed that human relation with and response from at least some of their enemies, was possible. As another example, Buber met T.S. Eliot, who was reputed to be antisemitic. A friend of Buber’s expressed surprise that Buber had interacted peacefully with T.S. Eliot. Buber replied, “When I meet a man … I am not concerned about his opinions but about the man himself” (Friedman, 2013, p. 36). To many people, these types of acts may seem strange and even morally questionable, but each one of these hints at a habit of I-Thou relations.

Some parallels to Buber’s I-Thou ideas exist in psychology. For example, Carl Rogers said his most effective therapeutic moments occurred when he experienced a relationship with “mystical subjectivity” (Rogers, 1955, p. 267), and he contrasted this to a more rigorous scientific approach. Likewise, May (1983) warned therapists that technical thinking about the client can hinder the therapist’s necessary full presence in the therapeutic relationship. Also, self-as-instrument theory has been interpreted as suggesting that an effective helper needs to engage in the psychological growth needed to become fully available to love others (Worth, 2017). Buber adds meaningfully to existing theory, in part because of his detailed description of the relationship with the precious other. This suggests that Buber can enrich existing psychological theory. In fact, a complete manuscript could be written to clarify Buber’s overlaps with and distinctions from existing psychological theory.

## 4. Relevance to suffering

Buber’s ideas can be applied to suffering in numerous domains. The focus here will be on three: individual, community, and dyadic-therapeutic.

### 4.1. At the individual level: anti-reactive

Buber describes a disruptive radical I-Thou dialogue that can occur with others, not only with loved ones or professional helpers. But how does this relate to suffering?

A common response to suffering might be to become preoccupied with cycles of painful ruminative cognitions that exacerbate suffering and damage relationships (Beck, 2019). For example, one could become preoccupied with self-blame which will have obvious negative psychological consequences. Another common response could be to become preoccupied with blaming the other. Even if that blame is accurate (as it was in Buber’s case of contact with the Nazis), a preoccupation could have unforeseen consequences. A preoccupation with blame of self or others could lead to a negative view of humans, both a low view of self and of others.

Buber argued that acting out spontaneous responses can lead to ruin, or, to use his analogy, the spark within each person will become perverted if that person spontaneously grabs at whatever is available in the world (Buber, 2002). Buber (1957) argued that a habit of mistrust is the root that hinders true peace between people, groups, and even nations. In a similar vein, Clifton and Meindl (2022) gathered evidence that many parents think they are doing their children a favor by warning them that the world is unfair, cruel, and that people are dangerous, and helping them develop a spontaneous defensive pose toward others. Those parents may think that teaching a cynical view of other people will protect the child from harm, that the warned child is more likely to avoid victimization. However, a negative view of humanity seems to produce ill-being (Clifton and Meindl, 2022; Helliwell et al., 2022) and predicts lower future pay increases (Stavrova and Ehlebracht, 2016) and societal problems (Elgar and Aitken, 2011; Helliwell and Wang, 2011).

I-Thou can disrupt these patterns of thinking. One cannot focus on catastrophizing or blaming self or blaming others or cynicism when experiencing dialogue entirely in the present, or when ignoring past sins or future benefits. During those moments, one turns toward the other. The I-Thou state may not be spontaneous, especially when experiencing difficulty, but Buber calls for resistance against the spontaneous (Buber, 2002). Thus, the I-Thou will resist, at least for some moments, catastrophizing, self-blaming and other painful cognitions, whether the context be job loss, a relational breakdown, or a health problem. Those types of cognitions lie in direct contrast to the dialogical approach advocated by Buber. No one lives permanently amidst I-Thou, nor should they, but I-Thou banishes such cognitions, at least momentarily. This perspective fits well with Wong’s (2007, 2019) approach to suffering because Buber does not promise a quick fix or an eradication of suffering, but instead a new way of being that can empower one to turn back toward the world of I-It and even suffering as a changed person.

In the long term, Buber suggested that genuine dialogue allows one to be oneself. It allows one to find the particularity in themselves and to learn their unique potential for contributing to the world in a way no other person can (Buber, 2002). As Buber said, “I become through my relationship with Thou” (Buber, 1957, p. 17).

Consider the following research. Schwartz and Sendor (1999) trained some multiple sclerosis victims in skills of listening and dialogue. The trainees were neither therapists nor advisors, but instead sufferers themselves who were briefly trained in careful attention to, and conversation with, another person. These laypeople then contacted others with multiple sclerosis and provided nondirective listening and dialogue once per month. They arranged a 15-min monthly meeting per case for a total of 3–4 h of dialogue per helper per month. The whole purpose was to show the impact on their clients, to show the power of receiving attention. The results, instead, showed much larger effects for those trained in listening and dialogue, with improvements in life satisfaction and depression, along with qualitative findings suggesting improved self-acceptance, self-confidence, and a sense of transcendence. Surprised by their findings, Schwartz and Sendor (1999) invoked a two-stage model to explain their results. In this framework the suffering person turns “away from themselves and toward some other entity (e.g., talisman, amulet, healer, or abstract divine being” but then turns back to look at “themselves and their condition with shifted perspective” (p. 1,565). This two-stage model captures some elements of Buber’s view of dialogue. Dialogue is not a cure for suffering, but dialogue is the response Buber recommended. He believed dialogue changes the self, so one can return to more mundane I-It relations as a changed person.

Other empirical evidence also supports Buber’s idea that relations create change. The Harvard Grant Study of Adult Development, for example, tracked people from the 1930s until the death of many of the participants (Vaillant, 2004). When predicting years of physically healthy happy life after age 50, relationship quality showed stronger predictive power than some of the obvious predictors such as cholesterol level. Similarly, other research suggests that positive contact, even with strangers, contributes to well-being (Lange, 2021), with some of the support coming from well-designed randomized control trials (Sandstrom and Dunn, 2014; Sandstrom et al., 2022).

Buber’s emphasis on relations concurs with a considerable body of psychological research. There is, however, no simple handbook for achieving I-Thou relations. We believe that a central implication of Buber’s perspective here is a potential challenge to empirically-minded psychologists. Namely, although there is considerable evidence to support general statements about I-Thou’s importance, the very nature of Thou’s uniqueness precludes a formula or algorithm for this kind of connection. The science leads us up to the gate of genuine connection with a unique other—but by definition cannot tell us what we should expect to see or what we ought to do, other than in the most general terms. We can be reminded of the importance of appreciating the uniqueness of another but we cannot be instructed in advance of how their uniqueness is constituted.

I-Thou may be beneficial, but also challenging, and this is especially so when one lives in a social context that hinders status, or even simply blocks social connections. After all, how many people can sustain their best efforts to truly see others while rarely if ever being seen? I-Thou cannot simply be an individual commitment; rather, there is a call to build and maintain communities that leave space for I-Thou.

### 4.2. At the community level: promoting dialogue

For Buber, the right response to suffering is dialogue within true I-Thou relations. He never claimed this could end suffering, but instead seems to have believed that relations can be healing and can bring a new focus. Many people in society feel excluded, however; and indeed, divisions within society may feel insurmountable.

Many scholars have tried to bridge differences between people and groups. Unfortunately, the state of the art in prejudice reduction has been largely dismal. Long-term prejudice reduction after intervention is rare (Paluck et al., 2021).

Some interesting recent research, however, has shown promise (Hartman et al., 2022). Some of this work suggests that genuine listening can reduce animosity (e.g., Kubin et al., in press). A memorable example can be found in deep canvassing research (Broockman and Kalla, 2016), which involves canvassers going door-to-door seeking support for a political change, such as support for a new law increasing transgender rights. Rather than primarily sharing arguments, the canvassers began by asking the resident to rate their support for a legislative change to improve the rights of people who are transgender. Next, the canvassers asked the resident to share a story of a time when they were not accepted. Then, the canvassers listened closely without judgment to the resident’s story of not being accepted. Next, the canvasser briefly shared a story of a time when the political status quo regarding transgender rights created similar feelings in themselves or a peer. Finally, the canvasser asked the resident to again rate their support for the legislative change. The 10-min intervention created changes in policy attitudes still measurable after 3 months. The deep canvassing framework seems to create change and showed the power of even brief relationship building.

The deep canvassing technique manipulates others, and some might even describe it as Machiavellian, but Buber offers something to move beyond this concern. Specifically, he considers much more broadly the extent to which dialogue and community may be possible.

Buber saw I-thou relationships as potentially much more pervasive throughout society. He defined community as, “a common life that embraces differences” (Kramer and Gawlick, 2003, loc. 1,227). Buber held a deep belief in dissimilarity between humans, believing that every person is unique and unlike any who has ever lived (Buber, 2002). From this perspective, relationships leave the dissimilarities undiminished; instead, through relationship, people will become fully themselves (Kramer and Gawlick, 2003).

Thus, for Buber, relationship requires bridging distinctions rather than simply finding like-minded others. This contrasts with a community of affinity which is formed because its members are all likeminded and feel that they have many commonalities in different avenues, such as race, religion, or politics (Kramer and Gawlick, 2003). Buber’s essential message is to aim for a community of otherness, in which its members may not be likeminded and similar in many ways, but they share the common goal of caring and living in togetherness. A community that fosters I-Thou dialogues, to be fully whole and fully present, is a community that asks us to at least be capable of surrendering ourselves. Teaching Buber’s ideals may help people move toward a society of greater resilience and compassion, a society that responds better to members who suffer. Note that this is not simply a call for more collectivism—nor for more individualism. If anything, Buber seems to endorse an unusual blend of the two cultural value systems: a third way that avoids both a self-serving self-focus (hence is more communitarian) and the reduction of people to social roles (valuing them as individuals). Thou values the *individual other* and is not an individual-ism.

Valuing the individual other demands much more tolerance of moral variability than either individualism or collectivism, an idea that fits well with some work by Gaus (2012). Diverse moral convictions lead to conflict, but Gaus saw neither possibility nor need to eradicate diversity of moral convictions. Gaus saw moral diversity as something to be expected and managed. He believed effective leaders will anticipate and manage rather than expect to fully eradicate the diversity of moral stances. How can one enter I-Thou when the other is inevitably distinct and different from the self? Rather than see this bridging of a divide as a problem, Buber framed it as a solution. Buber believed that “all real living is meeting” (Buber, 1958, p. 17). True dialogue is “turning toward the other” (Buber, 2004, loc. 508).

Some researchers have argued for recognition of shared traits or identities (e.g., common humanity) as a means to reduce animosity (Hartman et al., 2022; Voelkel et al., 2022). This strategy seems to have some value, but Buber took a different approach. His ideas fit better with Peter Singer’s claim that “Our best hope for the future is not to get people to think of all humanity as family-that’s impossible. It lies, instead, in an appreciation of the fact that, even if we do not empathize with distant strangers, their lives have the same value as the lives of those we love” (Singer, 2015, p. 80). Research by Mousa (2020) and Lowe (2021) provides further evidence that community building need not always require reduction of differences, but instead can involve collaboration across them. Wong (2011) has argued against a positive psychology that treats the good life as something to be achieved in a few easy exercises, as if people in extreme difficulty can follow these steps and have a happy and cheerful life. Buber calls people to a difficult task of relationship and true dialogue across differences.

Nonetheless, one concern with Buber’s focus on dialogue might be the relative neglect of oppression. Saguy (2018), for example, has argued that antipathy toward your oppressor is good, that it motivates social change. Dialogue and warm relations may sometimes disrupt motivation for change (Reimer and Sengupta, 2023). Admittedly, Buber offered little guidance for how to oppose oppression and how to change societal structures.

That said, Buber was hardly ignorant of oppression, as a Jew living in Germany in the 1930s. The Buberian orientation does not require a denial of reality, a denial of oppression, or a denial that struggle against others can be necessary. People suffer, and frequently that suffering is created or made worse by others. Buber never said to disavow struggle and effort to reform society, but he nonetheless saw the I-Thou as frequently possible and desirable.

Thus, Buber was advocating an antireactive approach to the life of suffering, but one that maintains space for struggle and activism. It might be difficult to advocate for I-Thou when one has suffered, not least when one has suffered from persecutory reduction to I-It. As an example, within Buber’s (2002) fictional story entitled *Heart-Searching*, the speaker gives advice to listen and be reflective yet is not someone who lived an easy life, but instead a victim of group-based persecution.

Parallel to our discussion of the individual level, we are again brought to a point where we can see the importance of living in these kinds of communities, but there cannot be a repeatable formula for how to establish them. There are some general principles; for example, that such communities are ethically important, potentially beneficial, demand commitment, and so on. Also, the descriptions of I-Thou that we provided can offer some sense of the destination. Nonetheless, different communities might pursue these ends in different ways and the I-Thou moments that emerge will themselves have their own uniqueness.

### 4.3. At the dyadic level: skilled healers

Buber seemed to realize the links between his thinking and psychotherapy. In fact, a central event of Buber’s life occurred when he failed to be fully present for a troubled student. Buber called this his “conversion” event (Kramer, 2019). Buber had been focused that morning on his own pursuit of mystical spiritual experience, so he provided only superficial conversation for the student who had arrived to talk. Later Buber heard from others that the student had been hoping for Buber’s help with a difficult decision. Without that help, the student went off to fight in World War I and died. Buber was troubled that his pursuit of a spiritual experience had distracted him from providing human interaction, thereby failing to meet a human’s need. After that, Buber turned away from mysticism and toward I-Thou encounters.

Buber explained his belief that dialogue could be therapeutic, among other places, in an essay entitled “Healing through meeting” (Buber Agassi, 1999). Also, he had sufficient interest in psychotherapy that he engaged in a public dialogue with Carl Rogers, which is available in transcript form (Buber Agassi, 1999). From those records, one can see Buber’s awareness that specialists at initiating encounter may have elevated ability to meet human needs. His thinking aligns in significant ways with theory and research on therapy.

When people are suffering from considerable psychological distress and they no longer wish, or no longer can, keep it to themselves, the first step in seeking help is often informal support. This advice can be sought from family members, romantic partners, friends, religious leaders, or other trusted members of the community. There is considerable evidence for the importance of social relationships as protective against prolonged suffering, and potentially sufficient even in cases when the suffering is acute (Kawachi and Berkman, 2001; Holt-Lunstad, 2018). Yet, there are times when this kind of help is insufficient: the person or their informal helper decide that a healer is required.

In *Persuasion and Healing*, Frank et al. (1993) present a transcultural model of healing practices. In their view, successful healing—which includes successful psychotherapy—involves four common elements: (1) an emotionally charged and confiding relationship between sufferer and healer; (2) special status and recognition of healing ability granted by the larger social context; (3) a clear rationale that is compelling to the sufferer; and (4) procedures and/or rituals that follow from the rationale. Although the authors noted that technique is by no means irrelevant, they argued that the success of all techniques requires the alliance. This view is supported by the evidence. Lambert (1992) studied four classes of common factors predicting therapy outcome and found that therapeutic alliance was second after aspects of the client and their external situation (e.g., a client meets an exciting new romantic partner, or suffers a death in the family, during a course of treatment). Therapeutic approach was ranked fourth, after hope and expectation; the lesser contribution of specific therapies has also been extensively documented by Wampold and Imel (2015). In a more recent meta-analysis, Flückiger et al. (2018) found that, after initial severity, no factor predicts outcomes better than therapeutic alliance.

How, then, does a therapist establish such an alliance? Therapist empathy, originally described by Rogers (1957), is routinely the strongest predictor of client progress across therapeutic approaches (Watson et al., 2002); meanwhile, lack of empathy consistently predicts negative outcomes (Mohr, 1995; Paulson et al., 2001). Unconditional positive regard, also first identified by Rogers (1957), also has support (Orlinsky and Howard, 1986; Orlinsky et al., 1994; Farber and Lane, 2010). Evidence for congruence is, however, decidedly more mixed (Cain, 2010). The centrality of empathy and regard do give us something with which to start. We are then confronted, however, with a narrower version of the question that started this paragraph: how does a therapist demonstrate empathy or regard?

The evidence supports several ways for therapists to enhance empathy: (a) ensure good eye-contact while maintaining a concerned expression; (b) lean forward while nodding the head appropriately; (c) maintain a vocal tone that indicates interest and emotional engagement; (d) communicate clearly; and (e) use emotional language (Watson et al., 2002). There is a problem here, however—and Buber helps us to see it. One cannot simply follow a set of instructions in order to emulate empathy, not least as there is also evidence that rote or ingenuine responses impair therapy (Glass and Arnkoff, 2000). Clients also vary in terms of what they understand to be empathic interpersonal behavior (Bachelor, 1988). In any case, Buber points to something more fundamental: a true encounter with another *cannot* make sense as a set of instructions. The attempt to do so is antithetical to the goal.

The existential and humanistic psychotherapy theorists have written most extensively about the therapeutic encounter in these terms. For May (1958), “the grasping of the being of the other person occurs on a quite different level than our knowledge of specific things about him [or her]” (p. 38). In other words, the other person must become Thou. Schneider and Krug (2017) describe the aim of existential-humanistic (E-H) psychotherapy as the endeavor to deeply understand the subjective experience of each client and their suffering and doing so while avoiding diagnostic or other theoretical presuppositions: “the E-H practitioner attempts to stay as open as possible to the living, evolving person who may or may not conform to present categorization” (p. 22). The ‘It’ of a particular diagnostic label, with its particular facts—albeit useful in specific circumstances—pulls us away from the ‘Thou’ who sits in the room with us.

Some of these theorists have engaged directly with Buber’s work. Friedman (1993), who wrote a biography of Buber as well as books on existentialism and psychotherapy, outlined a theory of psychological development that is explicitly dialogical. Describing this approach as ‘healing through meeting’, he argues that when one is present for oneself while being open for another, the possibilities and constraints that emerge from this genuine relationship are then transferred to the self. Rather than self-actualization being a personal striving that might lead someone to seek this kind of human connection, it may instead be better understood as a consequence or even a by-product of these encounters. For these reasons, “a relationship of openness, presence, directness, and immediacy,” is essential to the therapeutic relationship (Friedman, 2001, p. 344). Several other writers in this tradition, with varying degrees of direct engagement with Buber, make similar claims (Yalom, 2002; Mearns and Cooper, 2004; Yontef, 2007).

The argument here is not that we should abandon science and follow Buber. Rather, Buber and his fellow-travelers remind us that, although science can lead therapists to the threshold of I-Thou encounter by documenting its effects, it cannot then tell us what to do, precisely, with a specific person. The evidence outlines these relationships, argues for them and justifies them, but cannot finally instruct us on the specific ways we should be, in the moment, with another person. Indeed, this is not a limitation peculiar to science. The experienced clinicians referenced above also cannot provide these specifics, neither can Buber himself. Instead, he tried to capture some of the essence of what it means to have a genuine relationship. He tried to capture this essence through specific examples, especially in his *Tales of the Hasidim*, and then later through the aspirational prose of *I and Thou* and other works.

Rather than abandoning science, we might instead transform our use of science by considering research findings under a Buberian lens. Confronted by a specific client, we might be tempted to think, in effect, *this person has disorder X; the research shows that you get the best symptom reduction if you use therapeutic technique Y with disorder X, so I will now implement technique Y as accurately as possible*. It is not necessary to reject the database, only to reconsider how it is used. Perhaps instead, we might think, *I want to help this person using something I have to offer that I know has helped many others, as shown in the research, and I hope to offer this to them in a way that helps to relieve the suffering of this specific person, in this specific context*. The research becomes part of what we have to offer to another person, rather than something we apply to that person.

Besides, although psychotherapy research might not tell therapists precisely what to do, it can certainly get them started. There may be a large number of different ways to interact with a client that are directly helpful to them; there is surely a much larger number of ways to fail as a therapist. A recurring finding in the psychotherapy process literature is the better outcomes consistently observed in some therapists compared with others. Anderson et al. (2009), for example, demonstrated that therapists showing a set of characteristics, including the warmth, empathy, and alliance-building that points toward seeing a client as Thou, have better therapy outcomes. Incredibly, trainees able to show these characteristics during an interpersonally challenging situation in the first few weeks of training had better client outcomes 2–3 years later (see also Schöttke et al., 2017). While we cannot perfectly imitate these people, they can certainly show us the way.

## 5. I-Thou too difficult? Nuance as middle ground

For some, the I-Thou ideal may sound unattainable. As when one sees a tall mountain, the height of Buber’s I-Thou peak may create volitional paralysis rather than desire to climb. We think a conceptualization of levels underneath I-Thou might help rectify this problem.

We begin at the lowest level of relation. Buber believed that hatred requires a focus on only part of a person: “Hatred remains blind by its very nature; one can hate only part of a being” (Buber, 1970, p. 67). If you see your neighbor as only a person who impedes your privacy, you can hate them, but if you perceive a more complete person with worries and concerns like yours, hate may dissipate. When you hate, you are turning away from the whole person and justifying hatred by attending to a subset of their personhood. This approach is definitely within the realm of I-It. At its most extreme this type of focus on one hateful trait or a hateful subset of traits might provoke denial of all sense of personhood in the other, dehumanizing them, such as by labeling outgroup members as cockroaches (Vaes et al., 2021).

Above that, Buber anticipated utilitarian relations such as conducting business or work or collaborating with others. I may recognize that if I complete my assignments, my teacher will give a grade, or if I complete tax forms, the tax officer will give me a tax refund, or if I lend books, my neighbor will share home repair tools. This trade with and use of others need not be evil or harmful, but use is in the domain of I-It. Like hate, it may involve recognition of only one aspect of a person.

I-Thou, in contrast, cannot rely on trait descriptors or social roles, but instead takes a holistic view. There exists a long distance, however, between seeing one or a few aspects of another and experiencing I-Thou. We suggest that Buber’s ideas can be extended by inferring a middle ground between seeing a small slice of a person and the holistic perception of I-Thou.

Between seeing a small slice a person and transcending perception of traits and roles, we suggest a middle ground of nuance. Nuanced perception recognizes a combination of qualities that are sometimes at odds with one another. When I’m being nuanced, I will realize that my stubborn co-worker is more than merely stubborn; they may also sometimes be a loving friend or hard worker or good citizen. I move toward the unbounded view by holding consciousness that the other is more than the immediately obvious traits. Like a cloud that is partially clear and derived from the subtle transformations of vapor to water, the nuanced figure is veiled with a fog of subtle elements which flow together both paradoxically and in unity. Nuance creates the expectations to look further into our slight differences and forces us beyond our assumptions and generalizations.

In *The Way of Man*, Buber reflects on the uniqueness of each person: “Every person born into this world represents something new, something that never existed before, something original and unique” (Buber, 2002, p. 9). Because humanity is comprised of individuals who are unique in many minute ways, people can be considered deserving of nuanced perception. Every experience we live is uniquely our own. It is impossible to have every person in the world feel and behave in the exact same way because of the immense variability in our environments, and so it becomes inevitable that we cross paths with someone whose values, opinions, and beliefs are dissimilar to our own. Different perspectives and ways of life embraced by different people will inevitably clash. Nuanced perception recognizes many aspects of the other, some positive, some negative, and will prompt hesitation to judge the whole person because it anticipates there are many more yet unknown aspects of the other.

Nuanced perception, though is still not the full I-Thou relation. The I-Thou involves turning toward the other and perceiving the other as a whole, not focusing on particular traits, but nuance may provide a middle goal, a useful starting point for approaching I-Thou.

## 6. Other barriers to Buber’s ideas

One concern is that Buber’s I-Thou approach can be too tolerant of evil. Buber lived during the holocaust, and some have criticized his high and hopeful view of humanity as untenable in a post-holocaust world. The Post-holocaust Jewish philosopher Emil Fackenheim believed that “Buber had a lifelong difficulty with the recognition of evil” which became apparent in Buber’s response to the holocaust (Fackenheim 1982, p. 195). Others, however, argue that the holocaust was truly unthinkable, and that Buber’s reluctance to think the unthinkable is no discredit to Buber (Lawritson, 2012). Also, the fact Buber wrestled deeply with the horror of the holocaust is evident through his post-holocaust writings. Furthermore, Buber left Germany in 1938, before the full extent of Nazi cruelty was evident, and from 1932 to 1938 he spoke courageously and clearly against the evils that were evident. Buber’s claims are not a call to avoid the social activism that leads to social change, nor is it a call to believe people are good or always trustworthy. Buber never claimed people were all good, but he did argue that every person is precious (Buber, 2002). This belief that others are precious and worthy of dialogue, when accompanied by realization that those others are far from perfect, could be protective. A positive but realistic view of others seems to promote well-being (Tweed et al., 2021).

Also, the religious nature of much of his writing can create barriers to entry for people wanting to learn about Buber. Separating Buber from his religious roots would be difficult. His orientation expresses a Jewish anthropology that views humans as in relation, one that also hews close to a Christian perspective in some ways. Indeed, he engaged deeply with Christian thought (Buber, 2016) and has been influential to Christian writers in turn. From this perspective, we come into existence because of others, and we share existence through others, and at no point are we separable. Specifically, he brings an emphasis on religion as relationship rather than on religion as a set of intellectual assertions, and an emphasis on religious identity that is found through participation with others in community. Even though religious ideas permeate his thinking, his ideas can nonetheless hold broad appeal. For Buber, much of suffering is coping with the hiddenness of meaning and hiddenness of God. How could Buber maintain a dialogical understanding of reality when the God with whom he believed he was in dialogue appeared so silent and inactive during the Holocaust? Buber (2016) explores this question in *The Eclipse of God*.

All people, religious or not, face difficulty with the hiddenness of purpose and meaning in life. The question is much the same for both religious and nonreligious. If I put forth effort, if I contribute to the community, does it serve a purpose? Does suffering serve a greater purpose? Buber presumed yes and expresses that through valuing of relationships and experiencing relationship on the level of the immanent, with other people. As he said, in *Pointing the Way*, Each person “you meet needs help, each needs *your* help … [and] even when you yourself are in need—and you are—you can help others and, in so doing, help yourself” (Buber, 1957).

## 7. Exploring further

If readers want to explore these ideas further, they could read psychologists who have explored related concepts such as encounter (Rogers, 1980), presence (May, 1983), love (Fredrickson, 2014), faith in humanity (Tweed et al., 2021), and transcendence of suffering (Wong, 2023a). One could also read about interventions that bridge differences (Broockman and Kalla, 2016; Mousa, 2020; Hartman et al., 2022; Kubin et al., in press). There could also be value in examining Urban’s (2023) lighthearted trade publication focused on helping nonacademics learn to bridging differences. One may also benefit by reading about concepts that contrast with I-Thou, such as dehumanization (Haslam, 2022). Knowledge of Buber’s ideas and language can help enrich our reading of these related topics.

A good place to start with Buber might be his classic I and Thou book. It is short, though admittedly cryptic in some sections. The translations by Kaufmann (Buber, 1970) and Smith (Buber, 1958) both have value. A simpler place to start might be with the Kramer (2019) and Kramer and Gawlick (2003) works on Buber which offer a fascinating combination of narrative, life applications, and technical insight into Buber’s ideas. Also, the transcript of Buber’s discussion with Carl Rogers offers a glimpse of Buber’s way of being (Buber Agassi, 1999).

## 8. Key insights

Buber’s ideas offer a response to suffering, a way to transcend suffering. To call his direction a solution to suffering would be in some ways a misnomer. He makes no claim for eradicating suffering. He instead gives a response focused on I-Thou relations and dialogue. The deeper underlying meaning of events and suffering in the world may seem concealed, but Buber nonetheless called for persistence in turning toward the I-Thou.

The I-Thou relation becomes clearer when considering eight descriptors: Construed, turned, exclusive, present, unbounded, reverent, impermanent, and transforming. The relation is at least partly a matter of construal, i.e., one’s own chosen perception of others. The relation involves turning, being open to relation, and open to being influenced. The relation is exclusive; other concerns recede during I-Thou. I-Thou is present-focused; past injuries and future concerns are set temporarily aside. The Thou is unbounded; the self realizes it cannot comprehend or control the Thou. The self feels a sense of reverence toward the wonder that is the thou; the other is precious. I-Thou is impermanent. No one can live continually in I-Thou, but one can experience it and be changed. One becomes more of one’s true self after experiencing I-Thou.

In the individual domain, Buber’s ideal creates a wave that flows against the current of some spontaneous responses to suffering. These responses such as preoccupation with self-blame or other-blame or catastrophizing may create a cycle of suffering, but I-Thou will disrupt these at least temporarily. He calls for two stages, turning toward the other to enter I-Thou relation and then turning back as a changed person (see also Schwartz and Sendor, 1999).

In the community domain, Buber calls for broad dialogue across difference. Buber’s response to suffering involves not merely professional caregivers or therapists, but dialogue within community, dialogue in the form of I-Thou experiences. Thus, one does not run from suffering, but within suffering one meets others and relates. His call fits with empirical research on social contact but goes beyond by not calling for relations that reduce differences, but calling for relations across differences, dialogue that enhances both relationship and distinctiveness of each partner. This type of relation is a prescription for moments when the meta-narrative of the world is hard to find.

In the dyadic-therapeutic domain, Buber suggests that healing-in-relationship can be salutary for both sufferer and healer. The therapist might be in a professional-client relationship, exchanging money for services (I-It). The therapist may have diagnostic impressions, and these impressions may point to specific interventions (I-It). Yet there is potential for a genuine relationship, for the therapist to use the generalities of science in a caring way with a specific person, and to move beyond those generalities to connect with that person.

Some similarities can be seen with Wong’s (2023a) call for development of an existential positive psychology that addresses suffering while also promoting well-being. He argued that by facing suffering and managing relations, suffering can be transformed and transcended and become a path to wholeness.

Within a psychology of suffering, Buber’s ideas deserve attention for their focus on coping with both suffering and the hiddenness of meaning by turning toward others as precious beings, deserving of reverence. This approach may not eradicate suffering, but it may diminish suffering, change the meaning of suffering, and may transform the self to courageously face and transcend suffering.

## Author contributions

All authors listed have made a substantial, direct, and intellectual contribution to the work and approved it for publication.

## Funding

This work was supported by a Douglas College Research Dissemination Grant.

## Conflict of interest

The authors declare that the research was conducted in the absence of any commercial or financial relationships that could be construed as a potential conflict of interest.

## Publisher’s note

All claims expressed in this article are solely those of the authors and do not necessarily represent those of their affiliated organizations, or those of the publisher, the editors and the reviewers. Any product that may be evaluated in this article, or claim that may be made by its manufacturer, is not guaranteed or endorsed by the publisher.
